# Cell-Free Antigens from *Paracoccidioides brasiliensis* Drive IL-4 Production and Increase the Severity of Paracoccidioidomycosis

**DOI:** 10.1371/journal.pone.0021423

**Published:** 2011-06-22

**Authors:** Karen A. Cavassani, Fabrine S. M. Tristao, Leandro L. Oliveira, Fernanda A. Rocha, Jaqueline O. Vancim, Ana Paula Moreira, Ana Paula Campanelli, Luciano A. Panagio, Cristiane M. Milanezi, Roberto Martinez, Marcos A. Rossi, Joao S. Silva

**Affiliations:** 1 School of Medicine of Ribeirao Preto, São Paulo University, Ribeirao Preto, São Paulo, Brazil; 2 School of Pharmaceutical Sciences of Ribeirao Preto, São Paulo University, Ribeirao Preto, São Paulo, Brazil; 3 School of Dentistry of Bauru, São Paulo University, Bauru, São Paulo, Brazil; Universidade de Sao Paulo, Brazil

## Abstract

The thermally dimorphic fungus *Paracoccidioides brasiliensis* (Pb) is the causative agent of paracoccidioidomycosis (PCM), one of the most frequent systemic mycosis that affects the rural population in Latin America. PCM is characterized by a chronic inflammatory granulomatous reaction, which is consequence of a Th1-mediated adaptive immune response. In the present study we investigated the mechanisms involved in the immunoregulation triggered after a prior contact with cell-free antigens (CFA) during a murine model of PCM. The results showed that the inoculation of CFA prior to the infection resulted in disorganized granulomatous lesions and increased fungal replication in the lungs, liver and spleen, that paralleled with the higher levels of IL-4 when compared with the control group. The role of IL-4 in facilitating the fungal growth was demonstrated in IL-4-deficient- and neutralizing anti-IL-4 mAb-treated mice. The injection of CFA did not affect the fungal growth in these mice, which, in fact, exhibited a significant diminished amount of fungus in the tissues and smaller granulomas. Considering that in vivo anti-IL-4-application started one week after the CFA-inoculum, it implicates that IL-4-CFA-induced is responsible by the mediation of the observed unresponsiveness. Further, the characterization of CFA indicated that a proteic fraction is required for triggering the immunosuppressive mechanisms, while glycosylation or glycosphingolipids moieties are not. Taken together, our data suggest that the prior contact with soluble Pb antigens leads to severe PCM in an IL-4 dependent manner.

## Introduction

*Paracoccidioides brasiliensis* (Pb) is a thermally dimorphic fungus that causes paracoccidioidomycosis (PCM), the most prevalent systemic mycosis in several countries of Latin America including Brazil, Argentina, Venezuela and Colombia. PCM represents the major cause of disability and death among young adult rural workers during their most productive stage of life [1]. In 2001 it was estimated that approximately 10 million people were infected [2], and it seems that the number of new cases have been diminished every year, even in areas with high endemicity. Infection occurs by inhalation of fungal spores or particles, which transform into the pathogenic yeast form after reaching the pulmonary alveolar epithelium [3], [4]. Yeast can either be eliminated by immune-competent cells or disseminate to other tissues through lymphatic and hematogenous routes, resulting in a wide range of clinical and immunological manifestations, that vary from asymptomatic, benign and localized to severe and disseminated forms [5].

The broad spectrum of clinical and pathological manifestations is dependent on the patient's immune response. It is known that patients with PCM often present a depressed cellular immune response [3], [6], [7], [8]. Classical studies demonstrated that benign forms of the disease are associated with low levels of specific antibodies and positive delayed-type hypersensitivity (DTH) reactions [9]. In this context, the resistance to fungal infections are related to T helper 1 (Th1)-type cytokines such as IL-12 and IFN-γ, while susceptibility has been linked to the preferential production of the Th2 type responses, including IL-4, IL-5 and IL-10 [10], [11], [12], [13], [14]. Furthermore, several factors may contribute to this process, such as host and pathogen genetic background, fungal load and virulence [15], [16], [17].

After Pb infection, the host can be exposed to complex fungal antigens, including proteins, glycoproteins, and glycosphingolipids [18]. Although some of these antigens have been identified [19], [20], [21], the characterization and purification of others as well as their effects on the pathology during an in vivo infection are not well defined. The main *P. brasiliensis* antigenic component is the exocellular glycoprotein gp43 [20], [22], [23], an immunodominant antigen for cellular immunity in humans and experimentally infected mice [24], [25]. In addition, gp43 is implicated in suppression processes, participating in evasion mechanisms during the installation of primary infection, inducing inhibition of phagocytosis, NO and H_2_O_2_ production by macrophages [23], [26]. Likewise, the antigens released from the surface of *P. brasiliensis* (CFA) possibly have immunomodulatory effects during the course of disease. Previous studies showed that CFA injection induced suppressor T cells, which repress cell-mediated immune responses against *P. brasiliensis* [27]. However, the immunosuppressive mechanism and the identification of the responsible soluble immune factors remain unknown.

In the present study, we explored the role of cell-free antigens from *P. brasiliensis* in the modulation of immune response. Our results showed that the inoculation of CFA prior to the infection with *P. brasiliensis* resulted in disorganized granulomatous lesions and diminished control of fungal growth. The severity of the disease was attributed, in part, to IL-4 production, in an IL-10 independent manner. Taken together, our data indicated that CFA play an important role in the modulation of the immune response which leads to the progression of primary infection in hosts.

## Materials and Methods

### Ethics statement

All animal protocols used in this study were approved by the Institutional Animal Care and Use Committee at the Sao Paulo University, Ribeirao Preto, Brazil (protocol 086/2006). Accordingly, experiments were conducted adhering to both institutional and national guidelines for animal research, and all possible steps were taken to minimize animal suffering in these experiments.

### *P. brasiliensis* isolates

Yeast cells of a highly virulent strain of *P. brasiliensis*, Pb18, were maintained in the yeast-form in semi-solid Fava Netto's culture medium at 37°C and used at the 7th day in culture. The fungal cells were washed in phosphate-buffered saline (PBS, pH 7.2), and counted in hemocytometer. The viability of fungal suspensions was determined by fluorescein diacetate-ethidium bromide staining [28]. High viability suspensions (≥90%) were used in this study.

### Cell-free antigens (CFA) preparation

*P. brasiliensis* CFA was prepared as described previously [29]. To isolate different antigenic CFA fractions, three treatments were used: (a) 1 ml of CFA was boiled for 10 min in order to destroy its biological activity [30]; (b) 3 ml was deglycosylated through affinity chromatography using a Con-A column (0.7×2.5 cm, 1 ml, Sigma), equilibrated with PBS, and the unbound fraction collected; (c) 3 ml was delipidated using three wash cycles in water-saturated n-butanol solution (1∶1). The aqueous phase was collected after centrifugation at 10,000 *g* for 1 min. Protein concentration of all preparations was assessed with bicinchoninic acid assay (BCA, Pierce Scientific, Rockford, IL) and stored at −70°C until use.

### Mice and experimental PCM protocol

We used pathogen-free inbred mice obtained from our Isogenic Breeding Unit. Groups of five mice, 6 to 8-weeks-old, were used for each period of infection. Male C57BL/6 wild type (WT) – which shows an intermediate resistance during PCM, IL-4- (IL-4^−/−^), and IL-10-deficient (IL-10^−/−^) mice were supplied with sterilized food and water ad libitum, and maintained under specific pathogen-free conditions in micro-isolator cages in the animal housing facility of the Department of Biochemistry and Immunology, Ribeirao Preto School of Medicine-USP. Mice were injected once per week (three times) by subcutaneous route with CFA (5 µg per inoculation) or with PBS (control). Two weeks after the last injection, mice were challenged intravenously with 5.0×10^5^ viable yeast forms of *P. brasiliensis* diluted in 100 µl of PBS (Figure S1A). For the in vivo anti-IL-4 treatment, mice were intraperitoneally inoculated with 0.5 mg/ml of rat IgG or purified IgG1 mAb against murine IL-4 (11B11, 100 µl) one week after the last CFA inoculation, and at days −1, 7 and 10 of Pb-infection (Figure S1B).

### Assay for organ CFU

To determine the growth of *P. brasiliensis* yeast cells in the lungs, livers, and spleens, the number of viable microorganisms was determined by quantitative counts of colony forming units (CFU). The organs from five mice per group were harvested at days 15 and 30 post-infection (p.i), weighed and homogenized mechanically (Ultra Turrax, Ika-Werke, Germany) in 1 ml of sterile PBS. The homogenate was diluted 10 times in PBS and 100 µl of this suspension was placed on brain heart infusion agar (Oxoid Basingstok, Hampshire, UK) supplemented with 5% (v/v) of inactivated fetal calf serum plus 5% of *P. brasiliensis* 265 culture filtrate. The plates were then incubated at 37°C and colonies counted 7–14 days later.

### *C. albicans* culture and infection

The isolate was grown in Sabouraud dextrose agar medium at 37°C for 18 h. Suspensions of 1.0×10^6^ yeast cells (100 µl) were injected intravenously via the lateral tail vein in CFA-treated- and control mice (as described above). Kidneys, spleens and livers were removed at day 14 p.i. and CFU was analyzed.

### Organ homogenate and cytokine quantification

At days 15 and 30 p.i. the lungs were harvested and homogenized in 1 ml of PBS with phenylmethylsulfonil fluoride (PMSF; Sigma, St Louis, USA) using a tissue homogenizer (Ultra Turrax T8, Ika-Werke Germany). The samples were centrifuged at 10,000 *g* for 10 minutes. Supernatants were collected and stored at −70°C until analysis by sandwich enzyme-linked immunosorbent assay (ELISA). The levels of IFN-γ and IL-4 were measured using commercial antibodies (Pharmingen, San Diego, CA, USA) according with previous assays performed in our laboratory [31]. Optical densities were measured at 450 nm, using a microplate ELISA reader (EMAX; Molecular Devices).

### Histopathology

The lungs were excised at days 15 and 30 p.i., fixed in PBS 10% formalin for 24 hours, dehydrated and embedded in paraffin. Five-micrometer sections of tissue were stained with hematoxylin and eosin (H&E) for analysis of lung lesions.

### Statistical analysis

Statistical analysis was performed using ANOVA comparing multiple groups or by the parametric Tukey-Kramer test for two group comparison (GraphPad software). p<0.05 was considered to indicate statistical significance. The results are expressed as the mean ± SEM.

## Results

### Prior CFA inoculation resulted in diminished control of fungal growth

**Figure 1:**
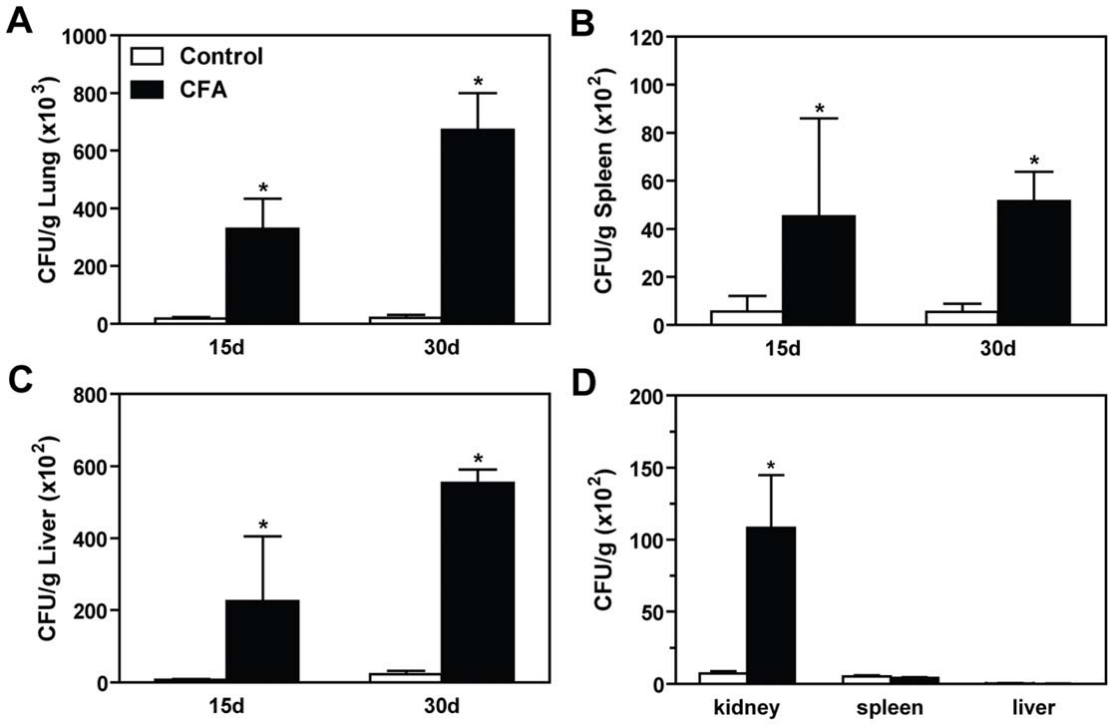
CFA induced severe granulomatous lesions and exacerbation of the PCM. Mice were sensitized with CFA (5 µg per inoculation) or PBS as control, once a week for three weeks via subcutaneous route. Two weeks after the third injection, mice were challenged intravenously with 5.0×10^5^ viable yeast forms of *P. brasiliensis* or 1.0×10^6^ *C. albicans*. The number of colony-forming units (CFU) was determined. At day 15 and 30 post-infection, the lungs (**A**), liver (**B**), and spleens (**C**) were removed from Pb-infected mice. The organs were weighed, homogenized in sterile PBS, serially diluted, and dispensed into Petri dishes containing brain–heart infusion agar. Plates were incubated at 36°C and colonies were counted 7–14 days later. (**D**) CFA-sensitized mice and controls were challenged with *C. albicans*. The fungal growth was analyzed in the kidney, spleen, and liver at day 14 post-infection. The scale bars represent the mean ± SEM of CFU per gram of tissue. *, p<0.05 compared with control group.

We first addressed the question if prior contact with CFA provided protection against a subsequent *P. brasiliensis* infection. Our results showed the CFA-inoculated mice showed significantly higher number of yeast cells compared with PBS-injected mice. The CFA treatment resulted in increased CFU of approximately 100 fold in the lungs, liver and spleen at days 15 and 30 p.i. compared with controls (Figure 1, A–C). Next, we evaluated if the effects of prior contact with CFA could extend to other fungal diseases, such as candidiasis. At day 7 post-infection the CFA-treated mice showed increased *C. albicans* recovery in the kidney, the major organ affected during candidiasis (Figure 1D). No significant differences in the fungal growth were found in livers and spleens (Figure 1D). These data indicate that prior CFA contact creates an environment that impairs the development of an appropriate immune response against fungal infection.

### The crucial role of IL-4 during CFA-induced immunosuppression

**Figure 2:**
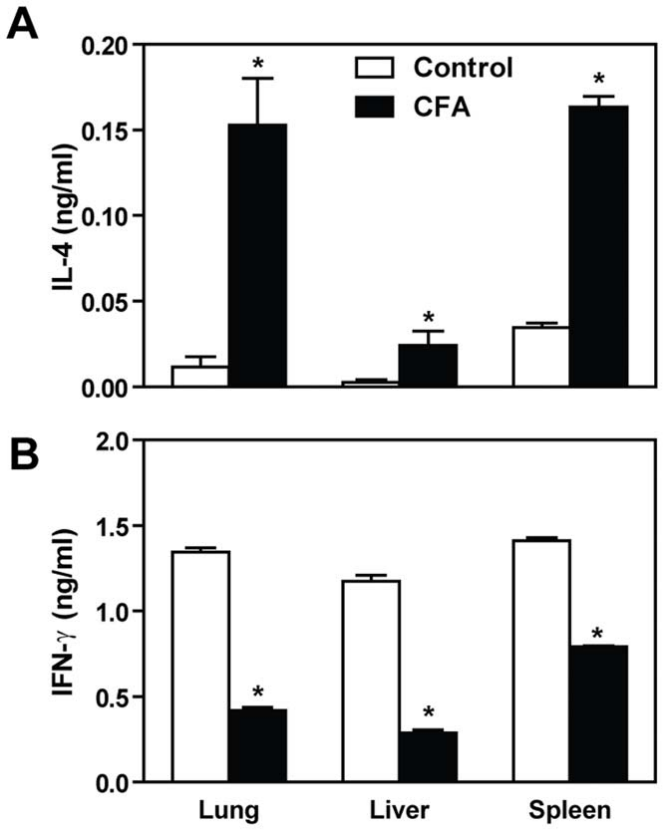
CFA induced high levels of IL-4 and diminished levels of IFN-γ. The levels of IL-4 and IFN-γ were measured by ELISA in the lung, liver and spleen homogenates from CFA-sensitized and PBS control mice at day 15 after *P. brasiliensis* infection. Data are means ± SEM from 5 individual mice. *, p<0.05 compared with control (not sensitized) mice. Data are representative of two independent experiments.

The Th1-type cytokines have protective effect during PCM, while a predominant Th2 response leads to increased susceptibility [10], [11], [12], [13]. In order to verify if the effects of prior CFA contact on experimental PCM is related to the skew of the immune response towards Th2, we measured the levels of IL-4 and IFN-γ in the lung, liver and spleen homogenates from CFA-treated- and control mice. Our data showed that at day 15 post-infection, the CFA-inoculated mice produced significantly higher amounts of IL-4 and diminished IFN-γ production in the indicated organs (Figure 2, A and B). When compared to control mice, the levels of IL-4 in CFA-treated mice were 13.07, 9.31, and 4.74 times higher in the lungs, livers and spleens, respectively (p<0.05). On the other hand, CFA-inoculated mice showed reduced amounts of IFN-γ in these tissues in comparison with control group (reductions of 3.2, 4.06 and 1.79 times, respectively, p<0.05).

**Figure 3:**
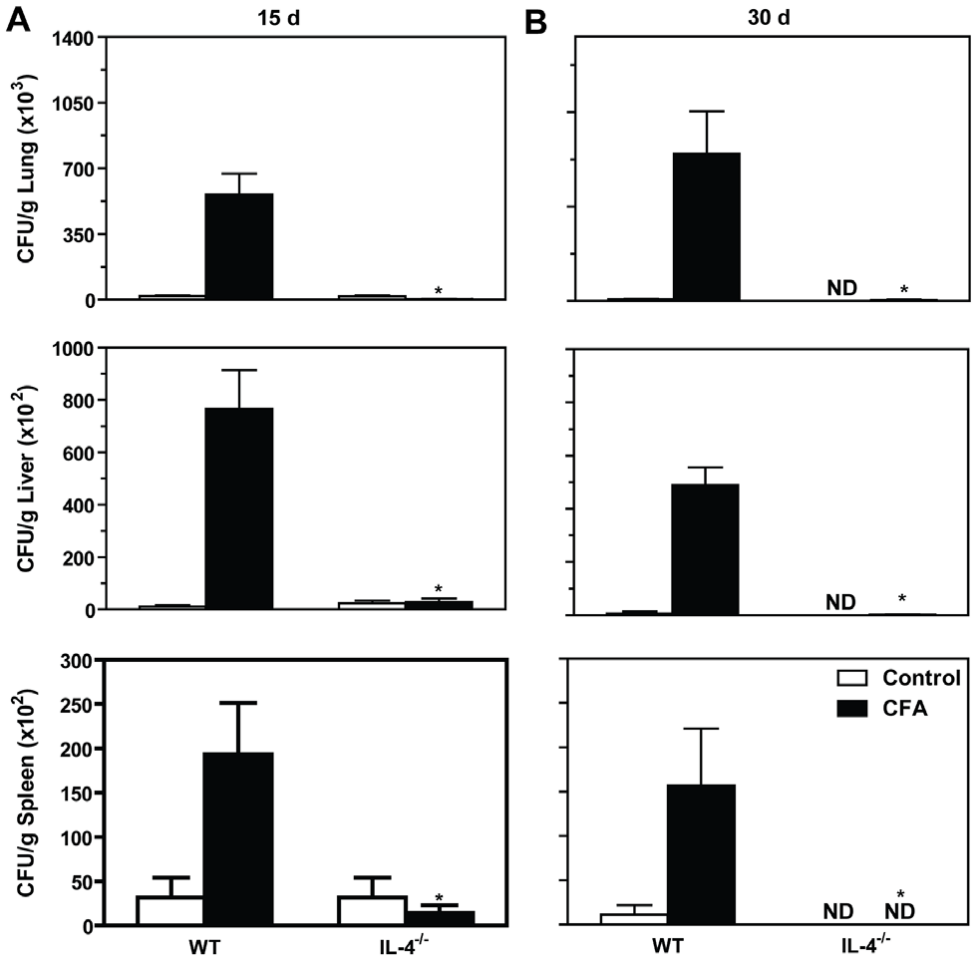
The immunosuppressive effects of CFA are mediated by IL-4. CFA-sensitized C57BL/6 (WT) mice, CFA-sensitized IL-4^−/−^ mice, control (not sensitized) WT and control IL-4^−/−^ mice were infected with *P. brasiliensis* and analyzed at 15 (**A**) and 30 (**B**) days post-infection as described in the legend to Figure 1. The scale bars represent the mean ± SEM of colony-forming units (CFU) per gram of tissue. *, p<0.05 in comparison with CFA-sensitized WT mice (n = 5–7). ND, viable yeast cells were not detected.

Next, we predicted that IL-4 would play an important role in the CFA- mediated immunosuppression. As shown in Figure 3A, at day 15 p.i., the prior CFA contact increased the fungal recovery in the lungs, liver and spleens from control WT mice in comparison with Pb-infected WT mice. On the other hand, IL-4^−/−^ Pb-infected- CFA-treated mice showed diminished amount of fungal cells in all analyzed tissues in comparison with WT-infected CFA-sensitized mice. Moreover, both WT and IL-4^−/−^ control Pb-infected mice showed similar CFU counts. As shown in Figure 3B, at day 30 p.i., CFUs were not detected in IL-4^−/−^ control Pb-infected mice while WT control mice had detectable, but low levels of yeast cells in the tissues. However, the increased fungal recovery mediated by the CFA-inoculation in WT mice was abolished in IL-4^−/−^ CFA-treated mice, suggesting that IL-4 is associated with immunosuppression and diminished control of fungal growth during experimental PCM after a pre-exposition to soluble *P. brasiliensis* antigens.

**Figure 4:**
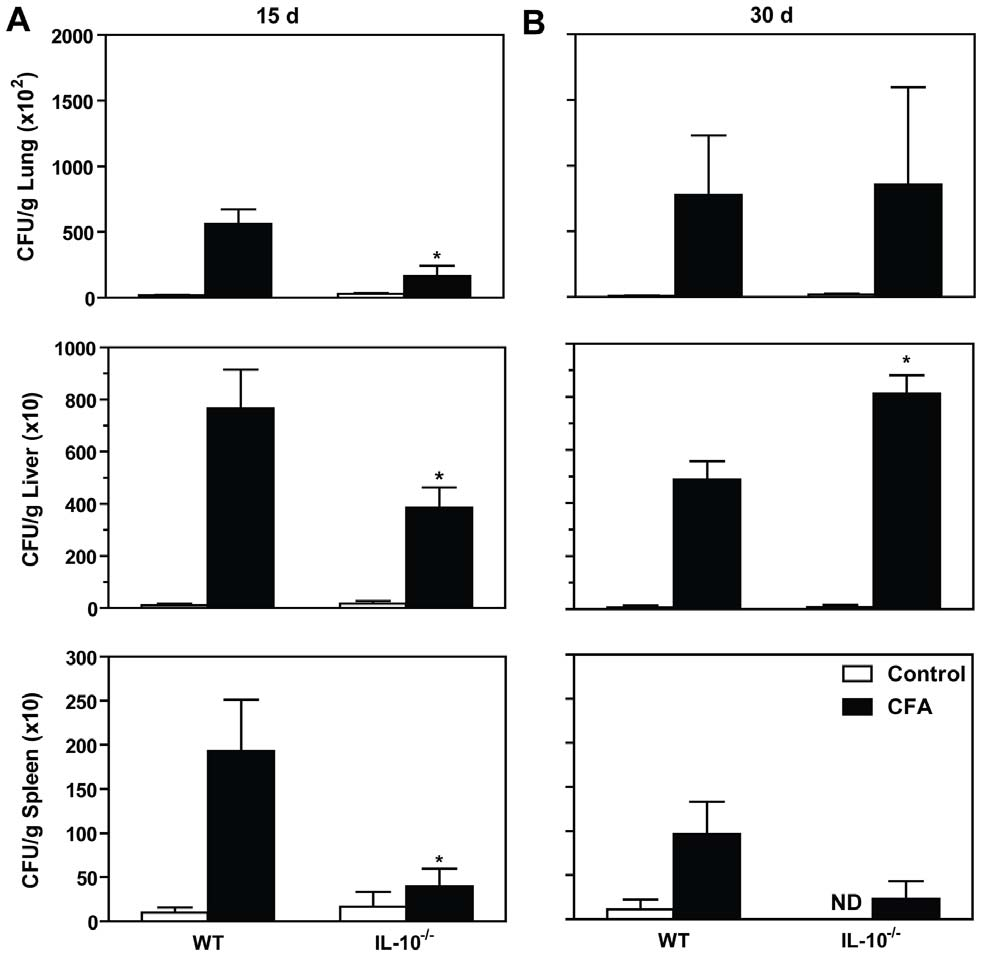
IL-10 is partially involved in the exacerbation of PCM mediated by sensitization with CFA. CFU of lung, liver and spleen homogenates from C57BL/6 (WT) or IL-10^−/−^ Pb-infected mice, sensitized or not with CFA is shown. Two weeks after the last CFA inoculation, mice were challenged intravenously with 5×10^5^ viable yeasts of *P. brasiliensis*. CFU counts were determined at (**A**) 15 and (**B**) 30 days post-infection. The bars represent the mean ± SEM of CFU per gram of tissue. ND, viable fungal cells were not detected.

We next asked if IL-10, a cytokine involved in the impaired activation of T cell immunity during PCM [32], was involved in the CFA-mediated exacerbation of the disease. In contrast to the results with IL-4^−/−^ mice, CFA-treated IL-10^−/−^ group showed similar CFU counting to CFA-inoculated WT mice in the lungs and liver at both 15 and 30 days p.i. (Figure 4, A and B). However, the fungal recovery in the spleen was decreased in CFA-treated IL-10^−/−^ mice compared to CFA-inoculated WT mice (p<0.05).

### CFA-treated IL-4^−/−^ mice showed less severe lung pathology during experimental Pb-infection

**Figure 5:**
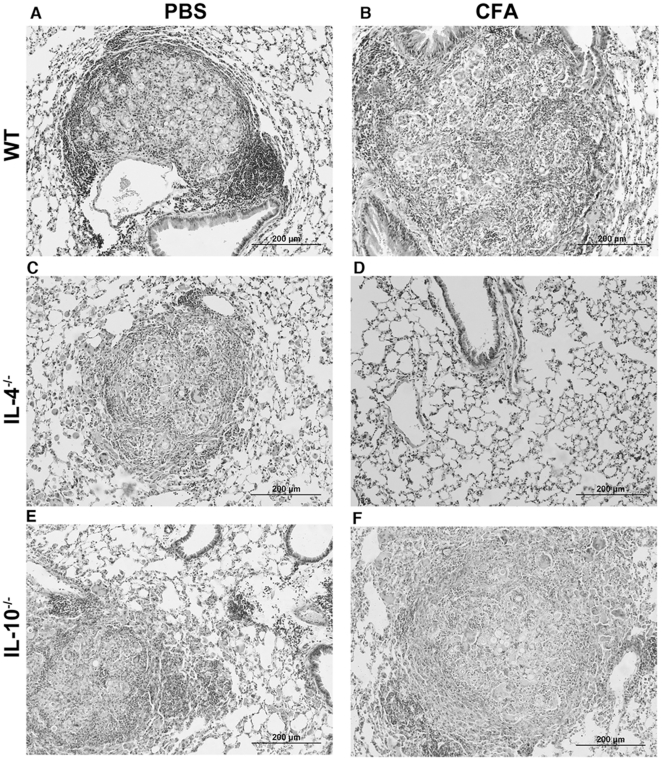
CFA-sensitized IL-4^−/−^ mice exhibited mild pulmonary lesions during PCM. Photomicrographs of granuloma lesions from (**A**) WT, (**B**) CFA-sensitized, (**C**) IL-4^−/−^, (**D**) CFA-sensitized IL-4^−/−^, (**E**) IL-10^−/−^, and (**F**) CFA-sensitized IL-10^−/−^ mice at day 30 post-infection. Bars: 200 µm. Lung sections were fixed in formalin, paraffin embedded, stained with H&E, and analyzed by light microscopy (Magnification, ×20).

Thirty days after Pb infection, the lungs from WT control group showed smaller and more organized lesions when compared with CFA-inoculated Pb-infected WT mice (Figure 5, A and B). Moreover, the pulmonary parenchyma was disrupted and an increased fungal load was present after CFA-treatment. When we analyzed the lung pathology in the IL-4^−/−^ control group, we observed smaller and more compact granulomas associated with diminished fungal burden when compared with WT control group. Further, CFA-treated IL-4^−/−^ lung did not exhibit granuloma formation (Figure 5, C and D), and this was consistent with the absence of viable yeast cells (Figure 3B). The IL-10^−/−^ mice exhibited the same lung granuloma profile as the WT group (Figure 5, E and F).

### Both IL-4 deficiency or neutralization inhibited the immunosuppressive effects of CFA

**Figure 6:**
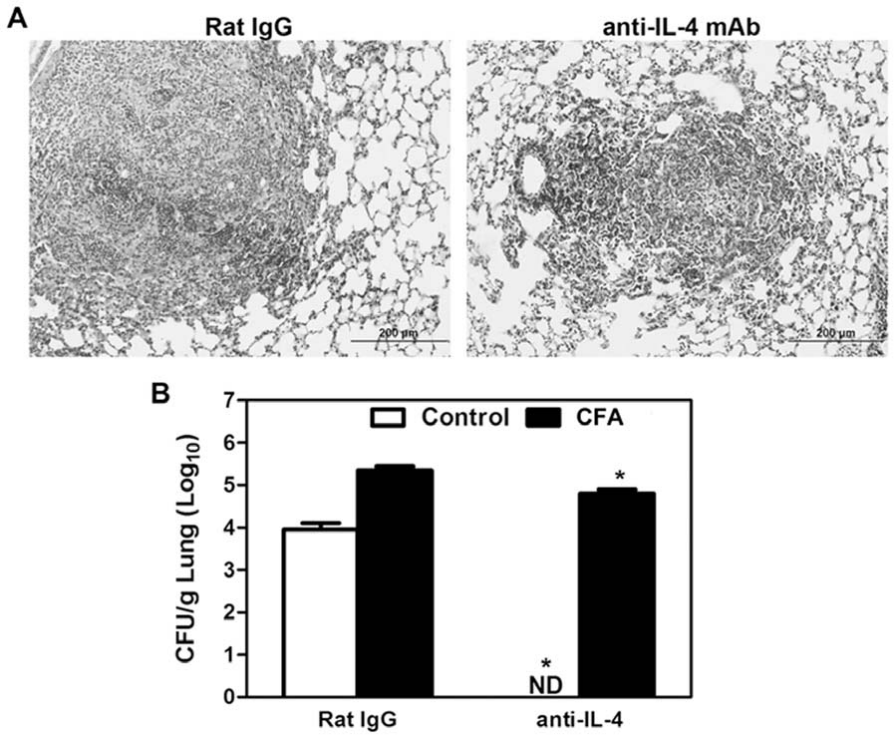
Anti-IL-4 markedly reduced pulmonary lesions and fungal growth in CFA-sensitized mice. Controls and CFA-sensitized mice were treated with anti-IL-4 mAb or rat IgG one week after the last CFA inoculation, and at days −1, 7, and 10 after *P. brasiliensis* infection. (**A**) Lung sections from CFA-sensitized mice treated with anti-IL-4 show smaller and more organized granulomas compared to granulomas in IgG treated mice. Hematoxylin-eosin stain was used; magnification, ×20. Bars: 200 µm. (**B**) Fungal growth recovery in the lungs from CFA-sensitized or control mice treated with anti-IL-4 or IgG is shown. The bars depict mean ± SEM of log_10_ CFU obtained from groups of 3–4 mice at day 15 after infection. Data is representative of two independent experiments. *, p<0.05 compared with rat IgG-treated mice. #, p<0.05 compared with unsensitized mice. ND, viable fungal cells were not detected.

Considering that CFA-immunossupression is IL-4-dependent, we next evaluated whether IL-4 needs to be present at the site and the moment of CFA-treatment. For this, WT mice were treated one week after the last CFA inoculation with anti-IL-4 antibody, which promotes the immunoneutralization of IL-4. Our results showed that the lungs from CFA-inoculated mice treated with anti-IL-4 mAb showed smaller granulomas, milder disruption of the pulmonary parenchyma and reduced infiltration of inflammatory cells in comparison with CFA- IgG-treated control mice (Figure 6, A and B). In addition, the treatment with anti-IL-4 mAb also resulted in a protective effect in non treated WT control mice. Furthermore, the IL-4 neutralization resulted in diminished number of viable yeast cells in the lungs from both CFA- and PBS-inoculated mice when compared to control IgG-treated mice (Figure 6C). These results suggest that IL-4, an important determinant in the severity of PCM in CFA-treated mice, does not contribute to the CFA action.

### Partial characterization of the fraction responsible for CFA effects

**Figure 7:**
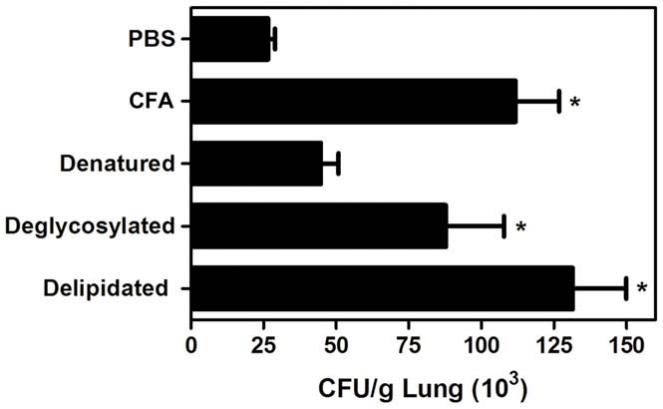
Protein fraction of CFA is required for immunosuppression during PCM. WT mice were sensitized once a week for three weeks with intact, denaturated, deglycosylated or delipidated CFA (5 µg per inoculation, subcutaneously). Two weeks after the last inoculation, the mice were challenged intravenously with 5×10^5^ of viable *P. brasiliensis* yeast cells. At day 15 post-infection, lungs were removed, weighed, homogenized in sterile PBS, serially diluted, and dispensed into Petri dishes containing brain–heart infusion agar. Plates were incubated at 37°C and colonies were counted 7–14 days later. The bars represent the mean ± SEM of CFU per gram of tissue. *, p<0.05 in comparison with PBS treated mice.

Proteins, glycoproteins, and glycosphingolipids are antigenic components of *P. brasiliensis* [18] that may modulate immune responses as has been described for gp43, the major *P. brasiliensis* antigen [22], [23]. After submitting our CFA preparation to a SDS-PAGE protocol, we could not identify gp43 (data not shown). In order to determine which fraction of Pb-antigen is responsible for IL-4-mediated immunossupression we performed a partial characterization of CFA. For this, WT mice were inoculated three times at weekly intervals with intact, denaturated, deglycosylated or delipidated CFA. Two weeks after the last inoculation, WT mice were infected with *P. brasiliensis*, and the CFU was recovered from the lungs at day 15 p.i. The number of fungal colonies observed in the pulmonary tissue from mice inoculated with intact, deglycosylated or delipidated exoantigen fractions of CFA were similar and increased in comparison to mice treated with denatured antigen (Figure 7). Together, these results indicate that CFA does not require glycosylation or the presence of glycosphingolipids to induce susceptibility during *P. brasiliensis* infection.

## Discussion

Vaccine approaches to infectious diseases are widely applied and besides the number of diseases that can be prevented by vaccines is growing, little is known about vaccination strategies against fungal infections. In the last years, several studies have been focusing in the *P. brasiliensis* experimental infection after immunization protocols. The vaccination with the recombinant protein PbHSP60 conferred partial protection in mice against a pulmonary PCM and allowed the identification of the cytokines and T CD4^+^ cells responsible for HSP60 protective properties [33]. In addition, low virulent fungal cells (via extensive DNA fragmentation) have been used to induce immunoprotection during experimental PCM. The prior contact of BALB/c mice with radioattenuated yeast cells of *P. brasiliensis*, which lost their virulence, promoted a long lasting protection associated with Th1 responses against highly infective yeast forms of *P. brasiliensis* [34], [35].

On the contrary, studies have demonstrated that soluble antigens from Pb lead to immunossupression during experimental PCM. Mice inoculation with the antigens released from the surface of *P. brasiliensis* (CFA), which can be recognized by receptors on the membrane of macrophages [36], [37], repressed the cell-mediated immune responses against Pb cells [27]. Considering that the immunosuppressive mechanisms and the identification of the responsible soluble immune factors remained unknown, in the present study we focused in the events involved in the immunoregulation triggered after a prior contact with CFA during a murine model of PCM.

We first evaluated the involvement of CFA in the modulation of granuloma formation, cytokine production, and control of fungal growth in Pb-infected mice. We showed that inoculation of CFA prior to infection resulted in severe lung pathology. The increased pulmonary CFU recovered from CFA-treated mice was accompanied by intense inflammatory infiltrate and severe granulomatous lesions. Moreover, CFA-treated mice showed granulomas containing central necrotic areas with diffuse cell distribution and a massive number of yeasts, which could be contributing to the intensity and the persistence of the inflammatory response in these mice.

The Th1/Th2 paradigm provides a basis for understanding T cell responses and is applicable to explain the resistant/susceptible pattern observed during experimental paracoccidioidomycosis. Cytokine studies, mainly in a pulmonary model of infection, have confirmed that Th1-biased immune response characterized by IFN-γ, TNF-α and IL-12 production, are linked with asymptomatic and mild forms of PCM [13], [14]. In contrast, a Th2 pattern has been associated with severe disease. Patients presenting the disseminated infection produce higher levels of type 2 cytokines, like IL-4, IL-5 and IL-10 [7], [38], [39].

In the present study, we described that the injection of CFA prior to infection induced a Th2 profile, increasing IL-4 levels in the lung, liver and spleen homogenates, and decreased the IFN-γ production when compared with only Pb-infected mice. Using IL-4^−/−^ mice and their IL-4-sufficient counterparts, we observed that the absence of IL-4 resulted in increased inflammatory reaction in the lungs as showed before [39], [40]. These data corroborates with elevated numbers of PMN cells in the early phase of infection (data not shown) and lower CFU counts in the pulmonary tissue. In agreement, previous studies demonstrated that the severity of PCM is mild in IL-4^−/−^ mice compared with WT group, with increased IFN-γ release in lung homogenates and enhanced fungicidal activity of alveolar phagocytes [39], [41], [42].

When we pre-treated mice with anti-IL-4 monoclonal antibody one week after the last CFA-inoculation, we observed that the in vivo IL-4-depletion impaired the suppressive effects of CFA. It implicates that IL-4-CFA-induced is responsible by the mediation of the observed unresponsiveness. Moreover, IL-4 favors the development of a Th1 rather than Th2 immune response. Because M2-macrophage activation is mediated by IL-4 and/or IL-13 (Th2-type cytokines), these macrophages are normally associated with immune responses that possess a Th2-skewed cytokine environment, as observed in parasite infections and allergic inflammation [43], [44]. Future studies should explore the role of these cells during *P. brasiliensis* infection.

Although IL-10 have an inhibitory effect during PCM by preventing fungal killing by IFN-γ or TNF-α-activated macrophages via a reduction in NO and H_2_O_2_ release [45], in the present study we did not find a crucial role for this cytokine during the immunosuppression mediated by CFA during the late phase of the infection. Using IL-10 deficient mice, we observed the absence of this cytokine resulted in a similar or increased lung and liver fungal growth at day 30 post-infection when compared with WT controls also exposed to CFA. At day 15, the absence of IL-10 resulted in decreased fungal growth in all tissues analyzed. No differences were found in only infected WT and IL-10^−/−^ mice. Since suppressive effects of IL-10 during paracoccidioidomycosis is well established [10], we propose that the presence of IL-10 during the first 15 days is essential to establish a initial negative regulation of immune cells via down-regulation of APC function or via Treg induction. However, the fungal persistence and excessive pathology induced by CFA at day 30 seems to be independent of IL-10, and it is IL-4 that is the pivotal cytokine driving the long-term immunossupression in CFA-treated mice. Also, the absence of IL-4 regulated the production of IL-10 during Pb infection [39]. Nevertheless, we cannot exclude the possibility that IL-10-related cytokines such as IL-22, IL-26, among others are compensating for the lack of IL-10 in IL-10-deficient mice [46].

The gp43, the major 43-kDa antigenic glycoprotein of *P. brasiliensis*, and the one single short peptide P10 from gp43 are able to promote increased protective immunity in mice when inoculated in the presence of adjuvant [24], [47]. When we fractionated our CFA preparation by SDS-PAGE, gp43 was not observed. However, it is possible that it could be present in low concentrations and, in this case, was not able to mediate a protective immunity. In addition, our CFA inoculation protocol was performed in the absence of adjuvant.

In order to determine which antigenic fraction of CFA, proteins, glycoproteins, or glycosphingolipids was responsible for the immunossupression, mice were inoculated with intact, denaturated, deglycosylated or delipidated CFA. Only the denaturated CFA treatment resulted in a significant decrease of viable yeast cells recovered from infected mice, suggesting that the immunossupression is associated with to a conformational protein presented in CFA, and not require glycosylation of the presence of glycosphingolipids and glycoproteins, like gp43 [23], [26] or paracoccin [36].

In summary, CFA amplify IL-4 production, which consequently impairs the Th1 response. Diminished control of fungal growth after CFA inoculation was dependent on IL-4 production. Moreover, the injection of CFA did not affect the course of infection in IL-4-deficient mice, suggesting the exacerbation of PCM after inoculation of CFA was mediated by IL-4 and not IL-10. In conclusion, our results suggest that CFA has an important role in the modulation of Th2-cytokines for the installation of primary infection in the hosts. Therefore, these data open new perspectives regarding anti-Th2 strategies, which might be considered in the treatment of PCM or other fungal infections.

## Supporting Information

Figure S1 **Experimental PCM protocol.** (**A**) Male C57BL/6 wild type (WT), IL-4- (IL-4^−/−^), and IL-10-deficient (IL-10^−/−^) mice injected once per week (three times) by subcutaneous route with CFA (5 µg per inoculation) or with PBS (control). Two weeks after the last injection, mice were challenged intravenously with 5×10^5^ viable yeast forms of *P. brasiliensis* diluted in 100 µl of PBS. (**B**) For the in vivo anti-IL-4 treatment, mice were intraperitoneally inoculated with 0.5 mg/ml (100 µl) of rat IgG or purified IgG1 mAb against murine IL-4 (11B11) one week after the last CFA inoculation, and at days −1, 7 and 10 of Pb-infection.
(TIF)
