# Supplementary figures and images for: Cell-Free Antigens from Paracoccidioides brasiliensis Drive IL-4 Production and Increase the Severity of Paracoccidioidomycosis
Source: PLoS One. 2011 Jun 22;6(6):e21423. doi: 10.1371/journal.pone.0021423 (PMC3120880; doi:10.1371/journal.pone.0021423)

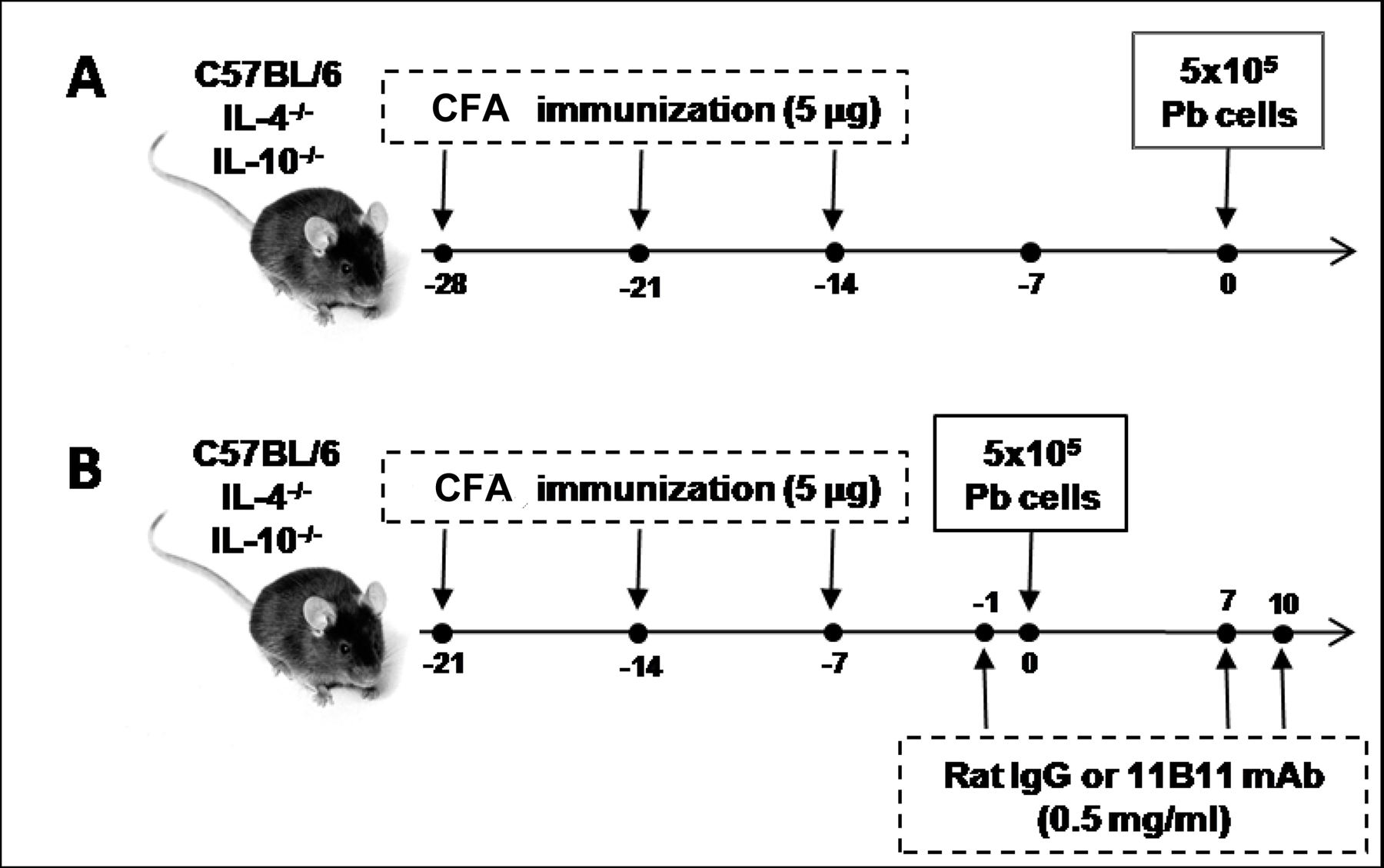

Supplement: Figure S1 — Experimental PCM protocol. (A) Male C57BL/6 wild type (WT), IL-4- (IL-4−/−), and IL-10-deficient (IL-10−/−) mice injected once per week (three times) by subcutaneous route with CFA (5 µg per inoculation) or with PBS (control). Two weeks after the last injection, mice were challenged intravenously with 5×10^5^ viable yeast forms of P. brasiliensis diluted in 100 µl of PBS. (B) For the in vivo anti-IL-4 treatment, mice were intraperitoneally inoculated with 0.5 mg/ml (100 µl) of rat IgG or purified IgG1 mAb against murine IL-4 (11B11) one week after the last CFA inoculation, and at days −1, 7 and 10 of Pb-infection. (TIF) [file pone.0021423.s001.tif]
